# Shelf‐life extension of *Fragaria × ananassa* Duch. using selenium nanoparticles synthesized from *Cassia fistula* Linn. leaves

**DOI:** 10.1002/fsn3.3336

**Published:** 2023-04-21

**Authors:** Arusa Aftab, Maira Ali, Zubaida Yousaf, Dalal Nasser Binjawhar, Sajjad Hyder, Zill‐e‐Huma Aftab, Zainab Maqbool, Zainab Shahzadi, Sayed M. Eldin, Rashid Iqbal, Iftikhar Ali

**Affiliations:** ^1^ Department of Botany Lahore College for Women University Lahore Pakistan; ^2^ Department of Chemistry, College of Science Princess Nourah Bint Abdulrahman University Riyadh Saudi Arabia; ^3^ Department of Botany Government College Women University Sialkot Sialkot Pakistan; ^4^ Department of Plant Pathology, Institute of Agricultural Sciences University of the Punjab Lahore Pakistan; ^5^ Center of Research, Faculty of Engineering Future University in Egypt New Cairo Egypt; ^6^ Department of Agronomy, Faculty of Agriculture and Environment The Islamia University of Bahawalpur Pakistan Bahawalpur Pakistan; ^7^ Center for Plant Sciences and Biodiversity University of Swat Charbagh Pakistan; ^8^ Department of Genetics and Development Columbia University Irving Medical Center New York United States

**Keywords:** antioxidant, nanotechnology, phenolics, selenium, shelf life

## Abstract

*Fragaria × ananassa* Duch. (Strawberry) fruit is susceptible to postharvest diseases, thus decrease in quality attributes, such as physiological and biochemical properties leads to decrease in shelf life. The objective of the present study was to check the effect of Selenium NP's and packaging conditions on the shelf life of strawberry (*Fragaria × ananassa* Duch) fruits. The shelf life was observed with 4 days intervals and examined for characteristics such as physiological weight loss, moisture content, percentage decay loss, peroxidase, catalase, and DPPH radical scavenging. The quality change of postharvest *Fragaria × ananassa* Duch. was monitored by the application of selenium nanoparticles (T_1_ plant extract in 10 mM salt solution, T_2_ plant extract in 30 mM salt solution, T_3_ plant extract in 40 mM salt solution, T_4_ distilled water; control) in different packaging materials (plastic bags, cardboard, and brown paper) at different storage conditions (6°C and 25°C). 10 mM, 20 mM, and 30 mM solution of sodium selenite salt, prepared from 1 M stock solution. Selenium nanoparticles were synthesized using *Cassia fistula* L. extract and sodium selenite salt solution. Polyvinyl alcohol (PVA) was used as a stabilizer. The nanoparticles were characterized through UV–visible spectroscopy and X‐Ray diffractometer (XRD). It was observed that the strawberry *Fragaria × ananassa* Duch. Treated with T_1_ (CFE and 10 mM salt solution) stored in plastic packaging at ±6°C showed the best physiological parameters and hence the treatment is recommended for storage without affecting the quality of strawberry fruit up to 16 days.

## Graphical abstract

Strawberry *Fragaria × ananassa* Duch. treated by T1 (CFE and 10 mM salt solution) stored in plastic packaging at ±6°C storage showed as the best recommended method of storage.

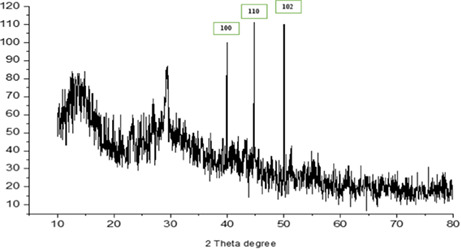

## 1 INTRODUCTION

Fruits and vegetables are an important food source. They are a rich source of carbohydrates, vitamins, proteins, and minerals. Fresh fruits and vegetables have always been in demand (Tian & Xu, 2022). Distribution and storage conditions badly affect the shelf life of fruits and vegetables. Loss of key characteristics, such as color, texture, sweetness, taste, and excessive moisture leads to shorter shelf life and a higher probability of customer refusal. So the concern is to keep food fresh and nutritious for a longer period of time. Packaging technology advancements and edible food coatings have demonstrated impressive outcomes in prolonging the shelf life of fruits and vegetables (Dias, 2012). Postharvest handling system of the distribution chain of fruits and vegetables is facing severe food spoilage problems due to poor postharvest management (Firdous, 2019). Strawberry (*Fragaria × ananassa*) is an important food crop belongs to the family Rosaceae. Globally, it is widely produced and consumed because of its pleasant flavor and nutritional value. Strawberry is a non‐climacteric fruits that should be harvested either before or during full ripening to receive the maximum nutrition, color, and taste (Velickova et al., 2013). Shelf life and the quality of strawberry fruits depends on their maturity stages. Harvesting before the optimum maturity time can enhance the shelf life but reduce the nutritive quality of the strawberry fruits. Whereas, fully ripened strawberry fruits exhibit high nutritive value with low lifespan. Hence, maintaining the equilibrium between maturity stage and nutritive quality is of utmost importance. The strawberry fruits with ¾ red surface are harvested commercially (Bahmani et al., 2022). Strawberry is well recognized for its high quantity of phenolics (flavonoids, anthocyanins, and ellagitannins), fibers, micronutrients (vitamin C, lutein, zeaxanthin, and choline), and minerals (Ca, Fe, Mg, K, Na, Zn, Co, and Se) (Afrin et al., 2016). Strawberry is liable to rot because of postharvest diseases, microbial contamination, and due to physiochemical changes (Azam et al., 2019). Strawberries have a relatively limited shelf life (approximately 1–2 days at room temperature and 3–4 days in the refrigerator) because of mechanical damage, excessive softness, rot induced by microbial development, moisture loss, and physiological abnormalities, during growth, harvesting, and storage (Panou et al., 2019). Several studies have documented effective postharvest management of strawberry fruits. The quality of strawberry fruits was maintained during storage period through the application of hot air and *Pichia guilliermondii* (Zhao et al., 2023). *Pichia guilliermondii* is documented to cause Fingernail infection (Zhang et al., 2020). Strawberry fruits packed in corrugated fiberboard packaging box treated with chlorine dioxide along with ethylene and moisture absorber were documented to have shelf life of 8.66 days (Dutta et al., 2023). The United States Food and Drug Administration (FDA, 2019) consumer alert documented that chlorine dioxide (CD) products were observed to have negative impact on human health (liver failure, dehydration, gastrointestinal ailments, etc.). Synthetic chemicals are posing serious threats to the environment and humans (Nicolopoulou‐Stamati et al., 2016).

Nanotechnology provides many benefits to the food industry in maintaining food taste, texture, sensitivity, quality, shelf life, and food safety. Nanotechnology is also used in food industries for preparation, packaging, marketing, and better yield and standard (Nile et al., 2020). The addition of nanoparticles into packaging materials provides quality and extension in the shelf life of fruits. The integration of NPs into polymers has empowered the development of more resistant packaging that is also cost‐effective (He et al., 2018). Application of proline‐coated chitosan nanoparticles on strawberry fruits increased the ascorbic acid content, phenolic content, total soluble sugars, enzymatic, and non‐enzymatic antioxidant activity during cold storage period (Bahmani et al., 2022). Arginine‐coated chitosan nanoparticles application on plum fruits reduced the weight loss, chilling injury, maintains the texture, ascorbic acid content, and enhanced the antioxidant activities (POD, CAT, APX, and SOD) during storage period at 1°C (Mahmoudi, Razavi, Rabiei, Palou, & Gohari, 2022). Postharvest application of glycine betaine‐coated chitosan nanoparticles enhanced the shelf life of plum fruits for up to 40 days during storage period at 1°C. Increase in DPPH activity, phenolic content, flavonoids, and anthocyanin was also observed in plum fruits during storage period (Mahmoudi, Razavi, Rabiei, Gohari, & Palou, 2022).

During the past 20 years, there has been a resurgence in green technology to improve the yield, nutritional, and postharvest quality of fruits and vegetables. Green synthesis of nanoparticles is an advanced method over other methods because it is simple, cost‐effective, eco‐friendly, and reproducible, and provides more secure products (Emamifar & Mohammadizadeh, 2015). Selenium (Se) initiates the biosynthesis of hormone and plays a defensive role in both adults and children. Se is reported to play a role in the biosynthesis of thyroid hormone; moreover, it is also involved in muscle movement, triggering an immune system against microbial infections and reproductive functions. An optimum intake of Se can eliminate the risk of cancer cell formation in humans. Se plays a defensive role in plants, as it reduces the damage caused by extreme climatic changes. It is known as a beneficial element for higher plants as it increases the production of secondary metabolites, antioxidant mechanism, and improves photosynthesis (Lanza & Reis, 2021). Selenium nanoparticles are getting increased popularity as a micronutrient and a dietary supplement in nanomedicine (Panou et al., 2019). Se nanoparticles are reported to have high bioavailability, significant bioactivities, and low cytotoxicity (El‐Ramady et al., 2016). Preharvest foliar application of selenium enhances the nutritional profile of kiwi fruits during storage period for 90 days at 1°C (Ghafouri et al., 2022).

The overall production cost in Pakistan is projected to be Rupees 100,000 per acre, whereas the income is rupees 200,000 per acre and the land area is 78 acres, with a yearly yield of 274 tons (Bangulzai et al., 2014). Therefore, improved infrastructure, postharvest production methods, and harvesting facilities can support it in domestic and foreign markets (Rajwana et al., 2016).

The present study was designed to observe the potential efficacy of SeNPs on physiochemical properties, antioxidant activities (enzymatic and non‐enzymatic), and storability in different conditions (different temperatures and packaging materials), Selenium nanoparticles were synthesized, characterized, and applied to postharvest strawberry fruits. The objective of the present study was to determine the effect of selenium nanoparticle and storage conditions on the shelf life enhancement of strawberry (*Fragaria* × *ananassa* Duch).

## 2 MATERIALS AND METHODS

The study was carried out in the Molecular Taxonomy Lab, Department of Botany, Lahore College for Women University, Lahore, from September to April 2022.

### 2.1 Plant collection for nanoparticle synthesis

Fresh leaves of *Cassia fistula* L. were collected, cleaned, air‐dried at room temperature, and then ground into fine powder with the help of a grinder. The powder was sieved and kept in an air‐tight container for further use (Bhalodia & Shukla, 2011).

### 2.2 Preparation of plant extract

About 10 g of plant powder was boiled in 100 mL of distilled water at 70°C for 20 min in a water bath. The supernatant was filtered using Whatmann filter paper No. 1 to remove the impurities. The supernatant was collected, re‐filtered, and centrifuged at 0.02236 *g* for 10 min and stored in jars for further use (Mulla et al., 2020).

### 2.3 Synthesis and stabilization of selenium nanoparticles

Selenium nanoparticles were synthesized using different concentrations of sodium selenite salt (10 mM, 20 mM and 30 mM) from 1 M stock solutions following the methodology of Lyubenova et al. (2015). A volume of 100 mL of different concentrations of sodium selenite salt solution (10 mM, 30 mM and 40 mM) were added into the 50 mL *Cassia fistula* L. aqueous leaves extract to make the reaction mixtures. For stabilization of nanoparticles, 0.1% of PVA was added and incubated at 37°C for 48 h to reduce the Na_2_SeO_3_.5H_2_O into Se‐NP's. The brown color of the mixture turned into dark brown at 48 h interval which confirmed the formation of SeNPs. Then the mixtures were allowed to cool at room temperature and centrifuged at 0.02236 *g* for 20 min. The supernatant was discarded and the pellets were washed thrice with distilled water and dried in oven at 25°C overnight (Chandramohan et al., 2019).

### 2.4 Characterization of selenium nanoparticles

Surface plasmon resonance of selenium nanoparticles was monitored by UV–visible spectrophotometer (Metash – Model V‐5800). UV–vis spectrometric readings were recorded at a scanning speed of 200‐800 nm wavelength range at the interval of 1 nm. To set the zero line, distilled water was used as a blank. The crystalline structure of selenium nanoparticles was confirmed by X‐ray diffractometer (XRD). XRD was performed in the 2 θ range from 10° to 90°. The crystalline size of green synthesized particles was determined using Debye Scherrer equation (D = 0.9λ/β cosӨ) (Jafarizadeh‐Malmiri, 2019; Logeswari et al., 2012).

### 2.5 Application of selenium nanoparticles on strawberry fruits

Strawberry fruits of uniform size, shape, and no physical or mechanical damage were purchased from the local market of Lahore, Pakistan and are transferred to the laboratory. The experimental design followed for the study was randomized complete split block design with four treatments, each treatment was run in triplicates. Selenium nanoparticles, 0.1% solution was prepared and 10 mL of each conc. was sprayed on 10 randomly selected strawberry fruits. Two different temperatures, room (25°C) and cold storage (4°C), three different packaging materials (polythene, paper, and cardboard) were selected for the application of nanoparticles. Treatments selected for the application of nanoparticles based on the production of nanoparticles included T_1_ plant extract in 10 mM salt solution, T_2_ plant extract in 20 mM salt solution, T_3_ plant extract in 30 mM salt solution, and T_4_ distilled water as control. Synthesized selenium nanoparticles were sprayed on the postharvested strawberry fruits bought from a local market in Lahore. Sprayed fruits were stored in three different packaging materials, such as polythene bags, paper bags, and cardboard packaging. Effect of SeNPs and storage conditions: temperature (6°C, 25°C) and packaging materials (plastic, cardboard, paper) on the shelf life of strawberry fruits were checked at 4 days intervals by observing the physiological weight loss, moisture content, decay percentage, enzymatic and non‐enzymatic antioxidant activities (Li et al., 2011).

### 2.6 Effect of SeNPs application on strawberry fruits

#### 2.6.1 Physiological weight loss, moisture content, and percentage decay loss

##### Physiological weight loss (PWL)

Physiological weight loss in strawberry fruits was calculated by comparing the original weight Wo (weight measured after applying treatment) and the weight (W) after 4 days interval (Hernández‐Munoz et al., 2008). The percentage physiological weight loss was measured by the formula:

$$\%\mathrm{PWL}=w_{0}-w/w_{0}\times 100$$

where, PWL = Physiological weight loss, *w*_0_ = Original weight, *w* = Weight measured after 4 days interval.

##### Moisture content

Empty petri dishes were weighed and 1.5 g of sample was added to each petri dish. These petri dishes were placed in an oven at 120°C for 2 h and then placed in a desiccator for cooling. Again, the weight of petri plates was noted and compared with the initial weight (Gharezi et al., 2012). The percentage of moisture content was calculated by:

$$\text{Moisture}\%=\frac{\text{Loss of weight}}{\text{Weight of sample}}\times 100.$$

where, Loss of weight = weight of petri dish and sample – weight of dried sample; Weight of sample = weight of petri dish and sample – weight of empty petri dish.

##### Percentage decay loss

Percentage decay loss was measured according to Ali et al. (2010). It was calculated by counting the number of fruits that decayed on 4 days' interval divided by the number of fruits included on the initial day of the experiment. The formula used to measure the percentage decay loss was (% decay loss = FD‐FI × 100), where FD = no. of decayed fruits and FI = no. of initial fruits.

#### 2.6.2 Increase in shelf life

The length of the extended shelf life was calculated by counting the number of days extended as compared with the control treatment (Moneruzzaman et al., 2008).

#### 2.6.3 Microbial susceptibility analysis

The antimicrobial analysis was performed on nano‐treated strawberry fruits by agar well diffusion method following the methodology of Daoud et al. (2015). Bacterial strains (*Pseudomonas fluorescence, Bacteroides ovatus*) and fungal strains (*Fusarium oxysporum, Aspergillus flavus*) were collected from the First Fungal Culture Bank of Pakistan (FCBP), Institute of Agricultural Sciences (IAGS) University of Punjab, Lahore.

#### 2.6.4 Enzymatic antioxidant activities

Enzymatic antioxidant potential of strawberry fruits was determined by peroxidase (POD) assay and catalase (CAT) assay (Kamińska et al., 2022).

##### Extraction of crude enzyme

A quantity of 0.2 g of strawberry fruit was ground under liquid nitrogen, then added 0.5 mL of 1 mM phosphate buffer saline (pH 7.0); 1 mL of 1 mM EDTA, 1 mL of 1 mM ascorbic acid, 1 mL riboflavin, 1 mL of 0.1% PVP. The samples were centrifuged at 0.0118 *g* for 15 min. The supernatant was collected and used for the determination of peroxidase and catalase enzyme activity (Kamińska et al., 2022).

##### Determination of peroxidase activity

Peroxidase activity was carried out by taking 1 mL of sodium phosphate buffer (pH = 7.0), 1 mL of H_2_O_2_, 1 mL guaiacol, and 0.5 mL enzyme extract to make the final reaction mixture, and absorbance was observed with UV–vis spectrophotometer (Metash – Model V‐5800) at 420 nm. Peroxidase activity was expressed in units of the enzyme that causes the absorbance increase of 0.1 per minute in U g^−1^ FW (unit per gram fresh weight).

##### Determination of catalase activity

A volume of 0.5 mL enzyme extract was taken and 1 mL of hydrogen peroxide was added and the activity was checked with UV‐visible spectrophotometer (Metash – Model V‐5800) at 240 nm resulting from the decomposition of hydrogen peroxide. One catalase unit was represented as the amount of H_2_O_2_ (μmol) decomposed by 1 g of tissue within 1 min (Kamińska et al., 2022).

#### 2.6.5 Non‐enzymatic antioxidant activity

Non‐enzymatic antioxidant activity of the strawberry fruits during storage period was evaluated by three assays named Total Phenolic content assay, DPPH Assay, and Total antioxidant assay.

##### Total phenolic content (TPC) assay

Total phenolic content was evaluated by following the methodology of Dewanto et al., 2002. A volume of 0.1 mL of each extract was mixed with 2.8 mL of 10% Na_2_CO_3_ and 0.1 mL of 2 N Folin–Ciocalteu (FC) reagent. The absorbance of the reaction mixture was measured at *ƛ*_max_ 725 nm by UV‐visible spectrophotometer (Metash – Model V‐5800). The results were obtained from a calibration curve (*y* = 0.005*x* + 0.047, *R* ^2^ = 0.998) of gallic acid (12.5–100 μg/mL) and represented as gallic acid equivalent (GAE) per gram dry weight.

##### DPPH (diphenyl picryl Hydrazyl radical) assay

DPPH radical scavenging activity assay was done by following the methodology of Kahraman and Feng (2021). A volume of 0.2 mL of each sample was mixed with 3.9 mL of DPPH solution and vortex. The samples were kept in dark for 15 min. UV‐vis spectrophotometer (Metash – Model V‐5800) was used for the measurement of absorbance values at *ƛ*_max_ 517 nm using DPPH as negative control and buthylhydroxytoluene (BHT) as positive control. The antioxidant activity was calculated as the following:

$$\text{DPPH radical scavenging activity}\;\left(\%\right)=\left(A_{c}-A_{t}\right)/\left(A_{c}\right)\times 100$$

where *A*_*c*_ is the absorbance of the control and *A*_*t*_ is the absorbance of samples.

##### Total antioxidants assay

Total antioxidant potential of the strawberry extracts was performed according to the methodology of Aftab et al. (2019). A volume of 0.1 mL of each extract was mixed with 1.9 mL of the reagent solution (0.6 M sulfuric acid, 4 mM ammonium molybdate, and 28 mM sodium phosphate). The incubation of the reaction mixture was done at 95°C for 60 min and was allowed to cool at room temperature. The antioxidant activity was expressed as the sample absorption at *ƛ*_max_ 695 nm using UV‐vis spectrophotometer.

### 2.7 Statistical analysis

Effects of different treatments, storage condition: temperature (6 and 25°C) of packaging materials on strawberry fruits' shelf life was subjected to two‐way analysis of variance (ANOVA) using IBM SPSS statistics 20 software and OriginPro 2022. Duncan's multiple range test was used to evaluate the differences between mean values deemed significant at *p* < .05 by using IBM SPSS 20 software.

## 3 RESULTS AND DISCUSSION

### 3.1 Synthesis of selenium nanoparticles

The color change from pale to reddish brown among all reaction mixtures within 48 hours indicated the synthesis of selenium nanoparticles. Surface plasmon resonance of all three reaction mixtures was determined by UV‐visible spectroscopy and crystalline structure by X‐ray diffraction (XRD).

### 3.2 Characterization of selenium nanoparticles

#### 3.2.1 UV–Visible analysis

**FIGURE 1:**
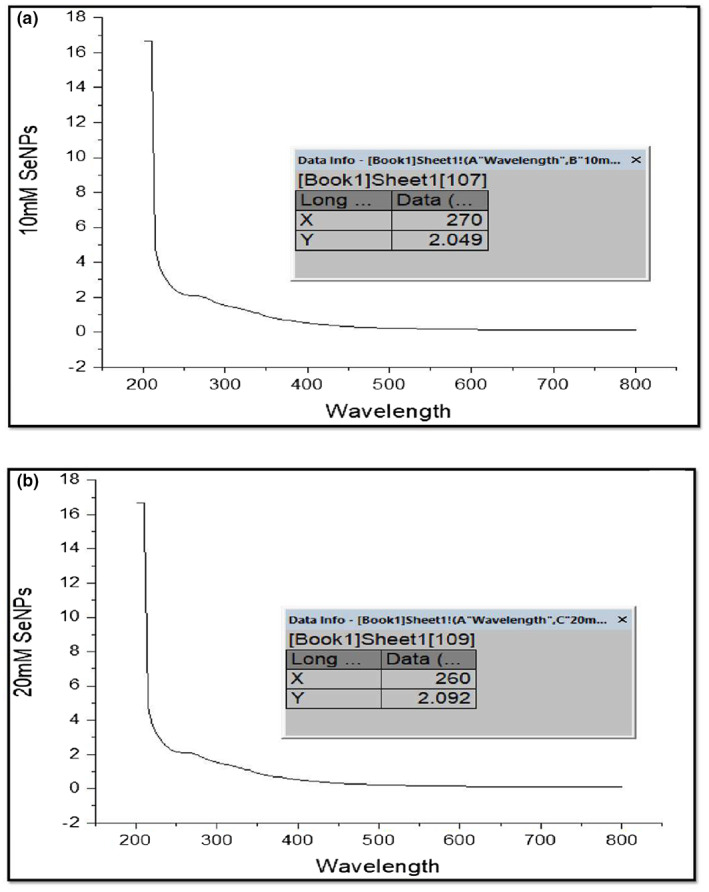
XRD analysis of SeNPs. (a) XRD analysis of 10 Mm SeNPs. (b) XRD analysis of 20 mM SeNPs. (c) XRD analysis of 30 mM.

The bioreduction of selenium ions in the solution was monitored by diluting a small amount of solution of 10 folds with distilled water and subsequently analyzed by the UV–vis spectrum periodically at different time intervals. Surface plasmon resonance (SPR) was observed at *ƛ*_max_ 270 nm (10 mM SeNPs), 260 nm (20 mM SeNPs), and 270 nm (30 mM SeNPs). It is reported in the previous studies that SPR of selenium nanoparticles causes a maximum absorption in between *ƛ*_max_ 200 and 400 nm. Selenium nanoparticles formed by *Aspergillus terreus* were observed to have SPR at *ƛ*_max_ 245 nm (Figure 1) (Afzal & Fatma, 2021). Ghaderi et al. (2022) observed the SPR at 298 nm.

#### 3.2.2 XRD (X‐Ray diffraction) analysis

**FIGURE 2:**
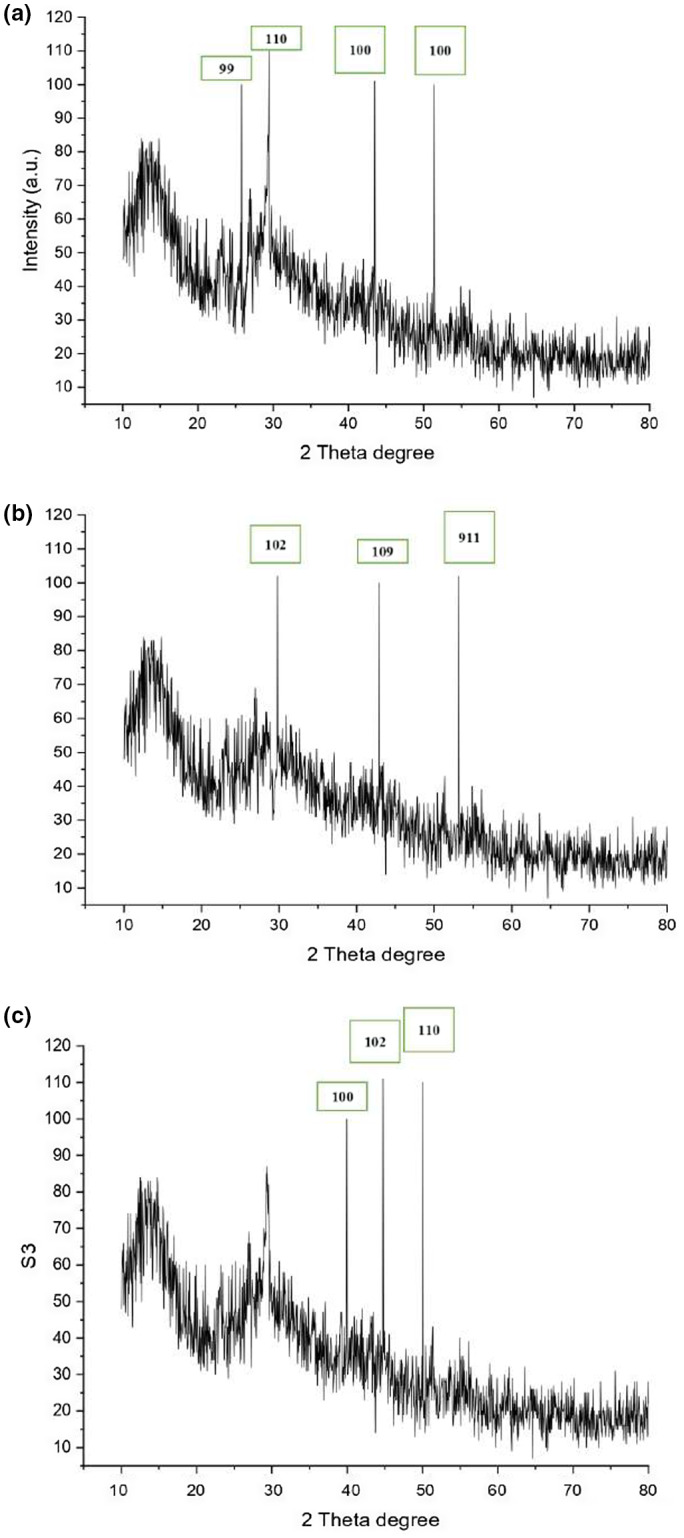
UV‐visible analysis of 10 mM SeNPs. (b) UV‐visible analysis of 20 mM. (c) UV‐visible analysis of 30 mM.

The crystalline structure and the average size of green synthesized selenium nanoparticles were analyzed with X‐ray diffractometer and are presented in Figure 2. Clear peaks of cubic phases at 25.8° (100), 29° (110), 43.61° (100), and 51.29° (100) were observed by 10 mM SeNPs; 20mM SeNPs showed clear peaks at 29.84° (101), 42.8° (109), 53.12° (110), and 30 mM SeNPs 29.33° (100), 39.9° (102), 44.6° (110), and 49.97° (111). The average crystalline size of SeNPs observed by Debye equation was 27 nm (10 mM Na_2_SeO_3_.5H_2_O), 34.5 (20 mM Na_2_SeO_3_.5H_2_O), and 88.9 nm (30 mM Na_2_SeO_3_.5H_2_O) (Figure 2). XRD pattern indicated that SeNPs were highly crystalline and spherical. (JCPDS file No. 00‐001‐0848). The peak intensity showed that the SeNPs were sized at the nanoscale. The study was similar to the outcomes of Ghaderi et al. (2022), in which SeNPs were synthesized from the aqueous extract of *Abelmoschus esculentus*.

### 3.3 Antimicrobial profile of SeNPs

**FIGURE 3:**
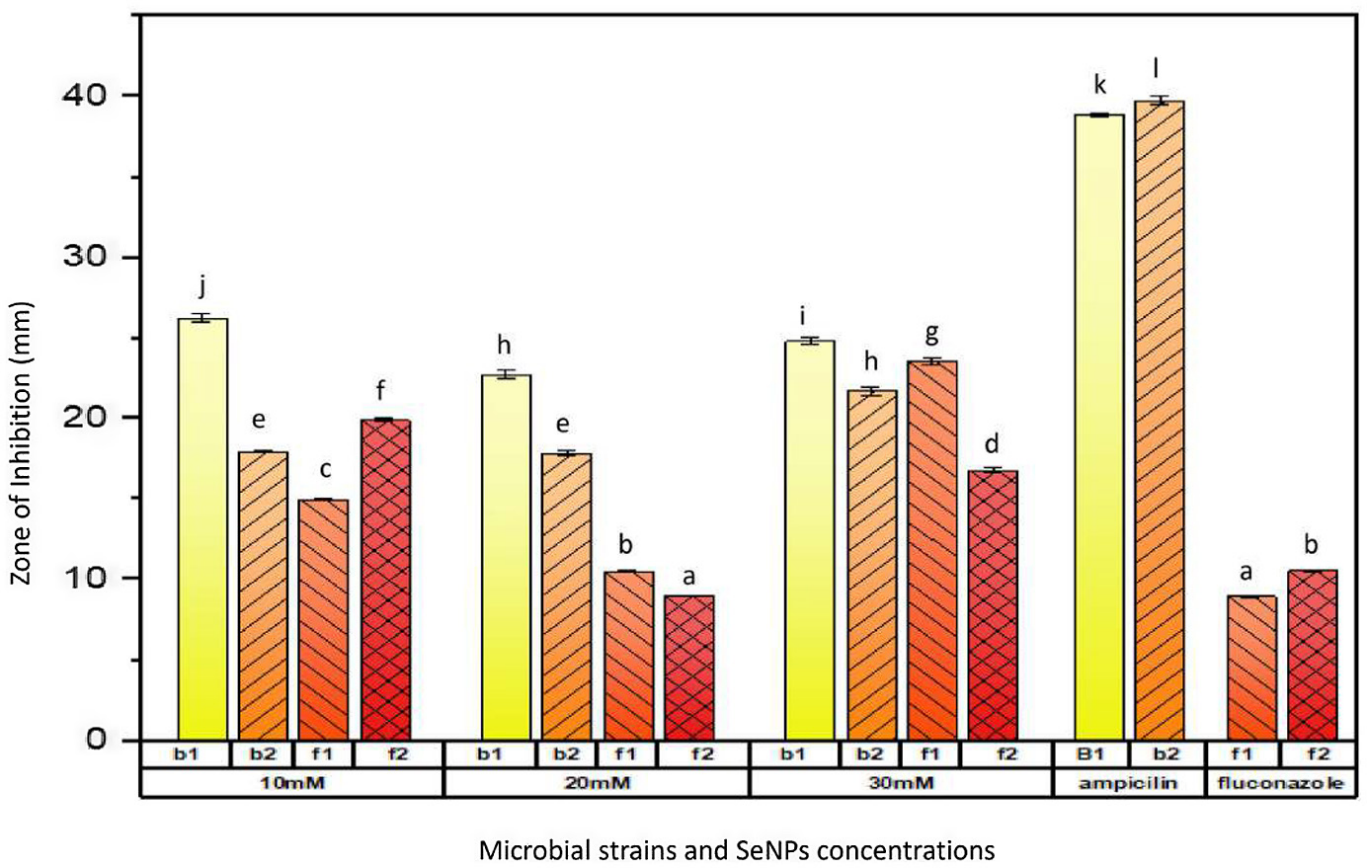
Antibacterial/antifungal potential of nanoparticles against different strains. b1 (*Pseudomonas fluorescence*), b2 (*Bacteroides ovatus*), f1 (*Fusarium oxysporum*), f2 (*Aspergillus flavus*); 10 mM (SeNPs); 20 mM (SeNPs); and 30 mM (SeNPs). Same letters on bars indicate the same difference between means, whereas different letters show the different mean differences between mean values at *p* < .05 according to Duncan's multiple range test.

The antimicrobial profile of green synthesized selenium nanoparticles was evaluated by an agar well diffusion method against two bacterial strains (*Pseudomonas fluorescence* and *Bacteroides ovatus*) and two fungal strains (*Fusarium oxysporum* and *Aspergillus flavus*); 10 mM SeNPs were observed to have a maximum zone of inhibition (26.8 ± 0.07 mm), whereas 30 mM SeNPs were observed to have a minimum zone of inhibition (22.2 ± 0.21 mm) against *P. fluorescence*. The 10 mM SeNPs also showed a maximum zone of inhibition (17.9 ± 0.06 mm) against *B. ovatus*a as compared with the highest concentration of SeNPs. Significant antifungal potential was observed for selenium nanoparticles against *F. oxysporum* and *A. flavus*. Maximum zone of inhibition, that is, 19.8 ± 0.17 mm was observed against *A. flavus* by 10 mM SeNPs, while 14.9 ± 0.45 mm was against *F. oxysporum*. (Figure 3). Cittrarasu et al. (2021) observed the inhibitory effect of green synthesized SeNPs against clinical pathogens (*E. coli* and *B. subtilis*). SeNPs synthesized from *B. subtilis* showed the zone of inhibition against 3 g‐positive bacteria (*Bacillus cereus, Staphylococcus aureus*, and *Listeria monocytogenes*), 3 g‐negative bacteria (*Escherichia coli, Salmonella typhi*, and *Klebsiella pneumonia*) and six fungal isolates (*Candida tropicalis*, *Candida albicans*, *Candida glabrata, Aspergillus flavus*, *Aspergillus fumigatus*, and *Aspergillus niger*) (Abdel‐Moneim et al., 2022). Hashem et al. (2022) confirmed that SeNPs synthesized from the aqueous extract of prickly pear peel waste exhibit antimicrobial activity against bacterial strains (*Staphylococcus aureus* ATCC 25923, *Pseudomonas aeruginosa* ATCC 27853, *Escherichia coli* ATCC 25922, *Bacillus subtilis* ATCC605) and fungal strains (*Cryptococcus neoformans* ATCC 14116, *Candida albicans* ATCC90028).

#### 3.3.1 Radical scavenging activity (DPPH assay) of SeNPs

Radical scavenging activity (%) of sodium selenite salt was evaluated as (44.2 ± 0.02) at the highest concentration (4.5 mg/mL). Standard butylatedhydroxytoluene (BHT) showed 74.1 ± 0.13 scavenging activity. The 10 mM SeNPs showed the highest scavenging activity 73.4 ± 0.1, which is close to standard. Minimum scavenging activity of 54.02 ± 0.04 was observed in SeNPs (20 mM). SeNPs (30 mM) showed the scavenging activity of 57 ± 0.2. These values were found closer to BHT (Table 1). Increase in DPPH scavenging % was observed with an increase in the concentration (0.25–1 mg/mL) of SeNPs, and this was observed in a study by Chen et al. (2022).

**TABLE 1:** DPPH free radical scavenging (%) activity of SeNPs.

Tested samples	1.5 mg/mL	3 mg/mL	4.5 mg/mL
10 mM SeNPs	73.8 ± 0.3^l^	64.7 ± 0.3^k^	53.7 ± 0.2^h^
20 mM SeNPs	33.86 ± 0.4^d^	46.86 ± 0.1^f^	54.8 ± 0.1^h^
30 mM SeNPs	57.3 ± 0.1^i^	50.3 ± 0.1^g^	28.56 ± 0.1^c^
Sodium selenite	44.5 ± 0.03^e^	24.92 ± 0.07^b^	14.82 ± 0.2^a^
Butylatedhydroxytoluene (BHT) (BHT)	44.3 ± 0.3^e^	60.2 ± 0.15^j^	74.1 ± 0.2^m^

*Note*: Letters in superscript indicated the statistical differences among treatments. Same letters indicate the same statistical difference between means values, whereas different letters indicate no statistical difference.

### 3.4 Effect of selenium nanoparticles on strawberry fruits during storage period

#### 3.4.1 Physiological weight loss, moisture content and percentage decay loss

Harvested fruits and vegetables are living commodities. They are characterized by high moisture content, metabolic rate, and firm texture. Water is the most important component of fresh produce. Water content is directly associated with macronutrients (proteins). Fruits and vegetables lose water through transpiration and respiration soon after harvest. And decrease in water content results in the reduction of weight and freshness (Singh et al., 2022).

**FIGURE 4:**
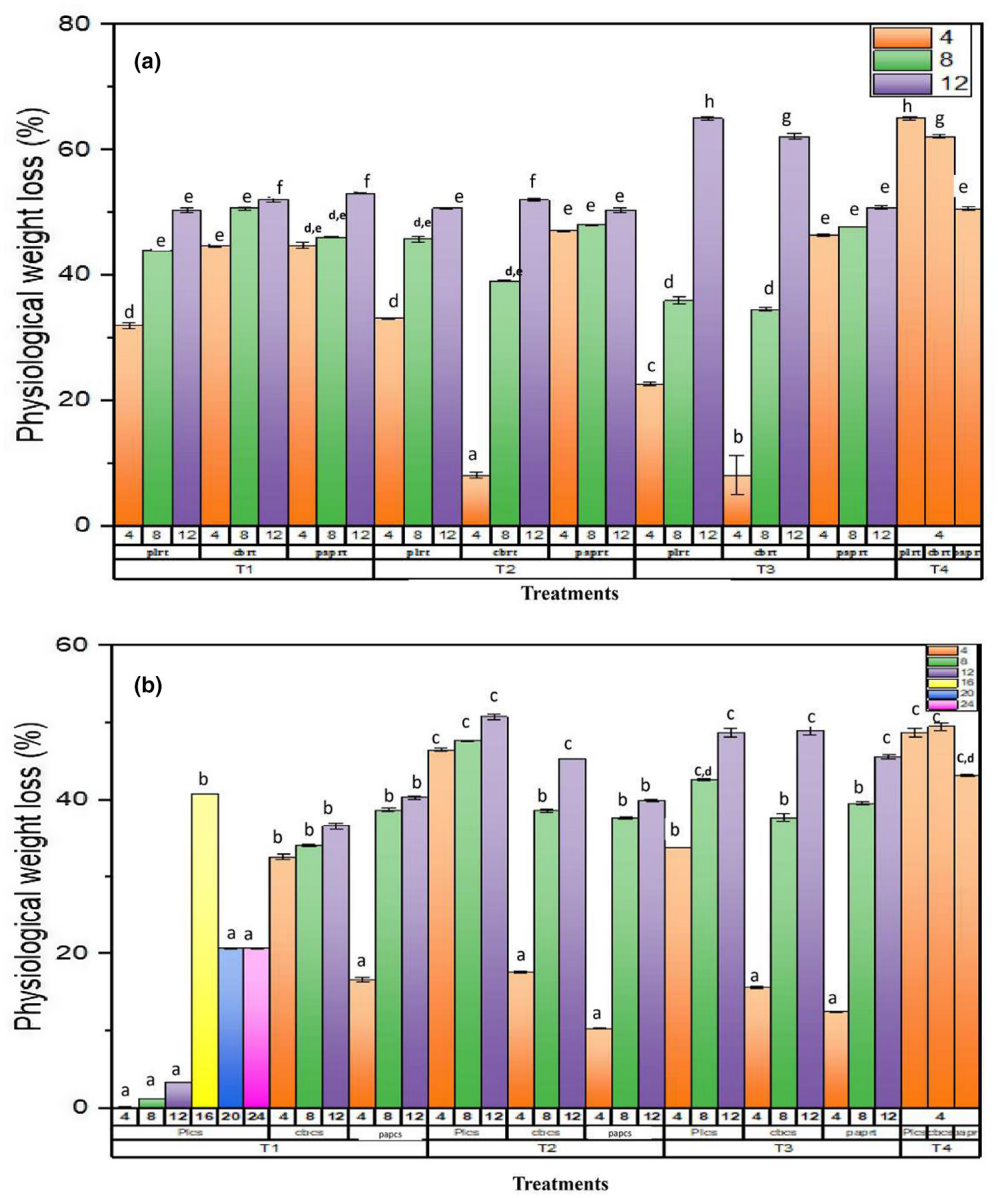
Effect of postharvest treatments and storage temperatures on physiological weight loss of strawberry fruit in different packing. (a) Storage period at 25°C; (b) storage period at 6°C. T1 (10 mM SeNPs); T2 (20 mM SeNPs); T3 (30 mM SeNPs); T4 (control); PL (plastic packaging); CB (cardboard packaging); pap (paper packaging); CS (6°C), and RT (25°C). Same letters on bars indicate the same difference between means, whereas different letters show different mean differences between mean values at *p* < .05 according to Duncan's multiple range test.

**FIGURE 5:**
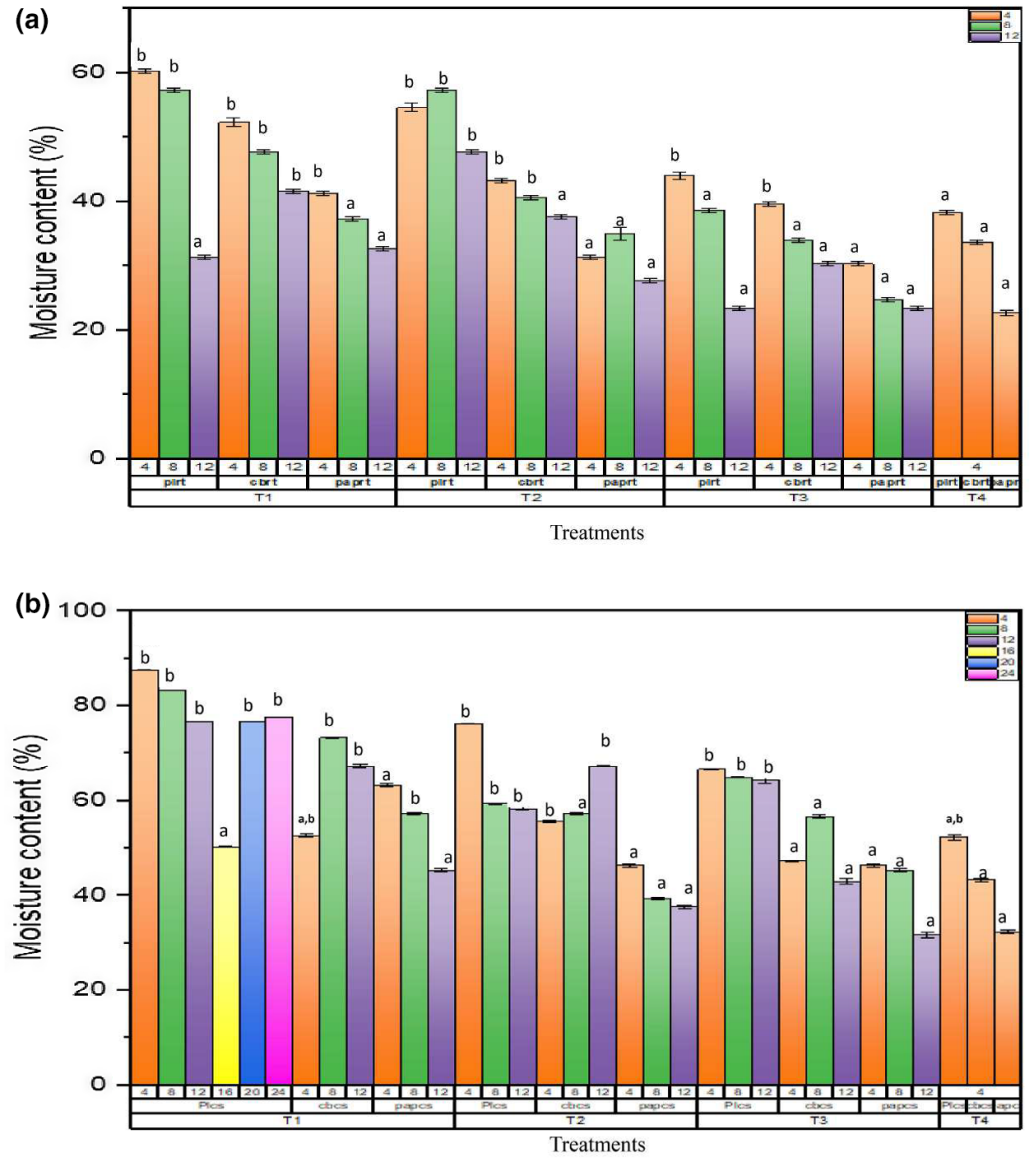
Effect of postharvest treatments and storage temperatures on the moisture content of strawberry fruit in different packing. T1 (10 mM SeNPs); T2 (20 mM SeNPs); T3 (30 mM SeNPs); T4 (control); PL (plastic packaging); CB (cardboard packaging); pap (paper packaging); CS (6°C); and RT (25°C). Same letters on bars indicate the same difference between means, whereas different letters show different mean differences between mean values at *p* < .05 according to Duncan's multiple range test.

**FIGURE 6:**
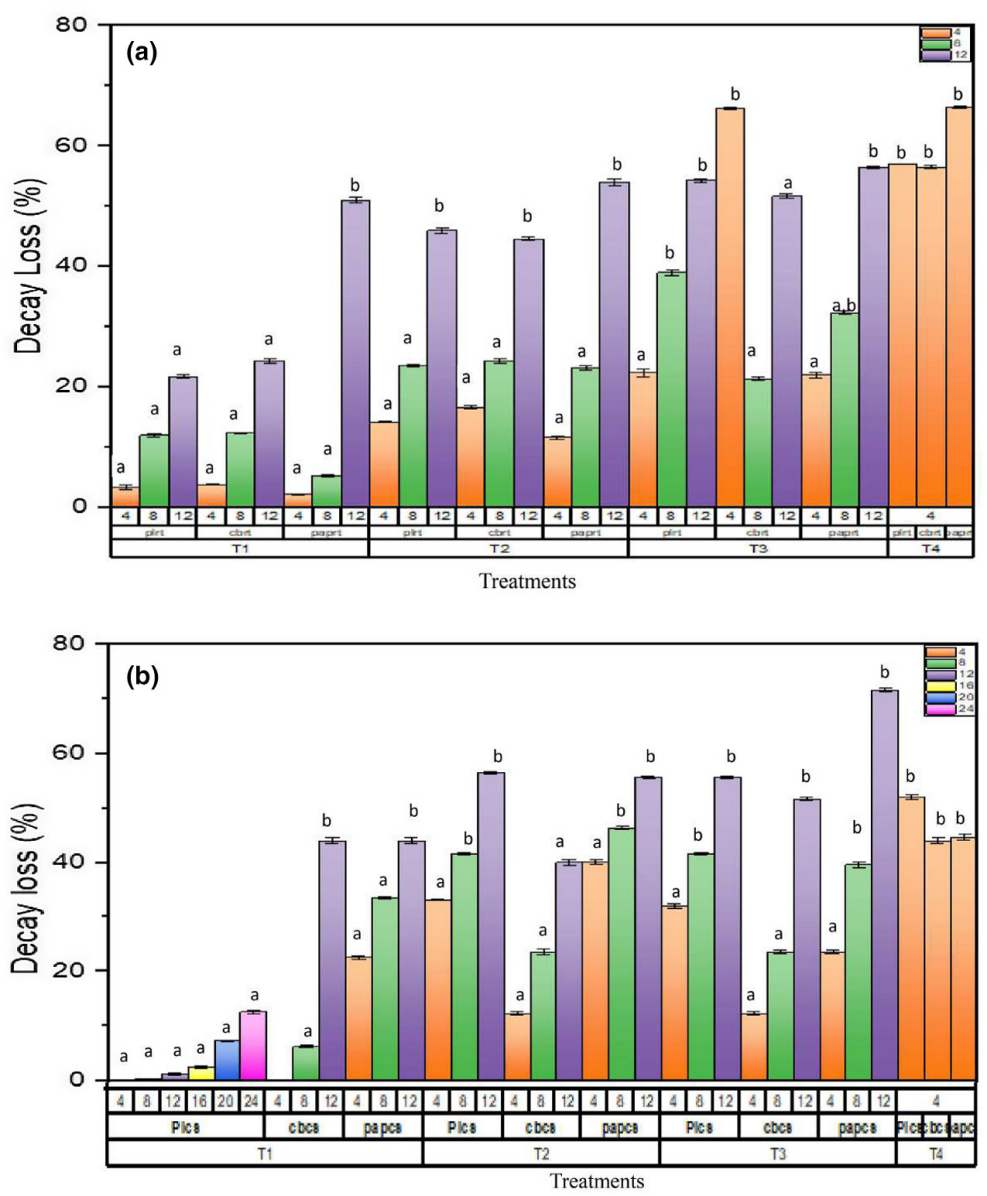
Effect of postharvest treatments and storage temperatures on percentage decay loss of strawberry fruit in different packing. T1 (10 mM SeNPs); T2 (20 mM SeNPs); T3 (30 mM SeNPs); T4 (control); PL (plastic packaging); CB (cardboard packaging); pap (paper packaging); CS (6°C); and RT (25°C). Letters in superscript indicate the same difference between means, whereas different letters show different mean differences between mean values at *p* < .05 according to Duncan's multiple range test.

The physiological weight loss (%), moisture content (%), and decay loss (%) 0 were determined during storage period (Figures 4, 5, 6). Among packaging materials, the highest weight loss (%) was observed in Cardboard and paper packaging at 25°C, whereas minimum weight loss (%) was observed in plastic packaging at 6°C. It is clear that significant decrease in weight loss (%) was observed in SeNPs‐treated strawberry fruits as compared with control (T4). Strawberry fruits treated with T1 was observed to have a minimum weight loss (%) in plastic packaging on day 16, that is, 3.31 ± 0.005 at 6°C. T3‐treated strawberry fruits were observed to have maximum weight loss (%) in cardboard packaging on day 12 at 6°C. The reduction in weight loss (%) of strawberry fruits was observed up to 8 days of storage period when subjected to the application of 50 ppm oregano essential oil stored at 7°C by Faozia et al. (2022).

During storage period, SeNPs‐treated strawberry fruits were observed to have higher moisture content as compared with control. Among the SeNPs treatment, T1 was observed to have higher moisture content on day 16 at 6°C as compared with control and other treatments (T1, T2, and T3). Similarly, higher decay loss (%) was observed in SeNPs‐treated fruits as compared with control treatments. Least decay loss was observed in T1‐treated strawberry fruits kept at 6°C. Comparison of both storage temperatures (6°C and 25°C), it was clear that the fruits kept at 6°C storage showed extended shelf life than that stored at room temperature. The reason behind this was that at cold storage, the process of ripening of fruit slowed and moisture loss was decreased. The findings were in correspondence with Saleh and Abu‐Dieyeh (2022). The study also documented the factors involved in the decay of strawberry fruits which include an increase in skin permeability for loss of moisture, an increase in respiration rate, and vulnerability to decaying organisms.

The senescence rate of postharvest fruits and vegetables is determined by respiration rate. Respiration involves a series of redox reactions that utilize carbohydrates and organic acids as substrates. Increase in respiration rate causes an increase in the metabolic rate of fruits (Du et al., 2022). Sang et al. (2022) observed the effect of temperatures on postharvest quality and antioxidant profile of *Zizyphus jujuba* Mill. cv. Dongzao during storage period. The study revealed that fruits stored at low temperature (0°C) were observed to have a decrease in respiration rate as compared with fruits stored at room temperature (25°C).

#### 3.4.2 Increase in shelf life

**FIGURE 7:**
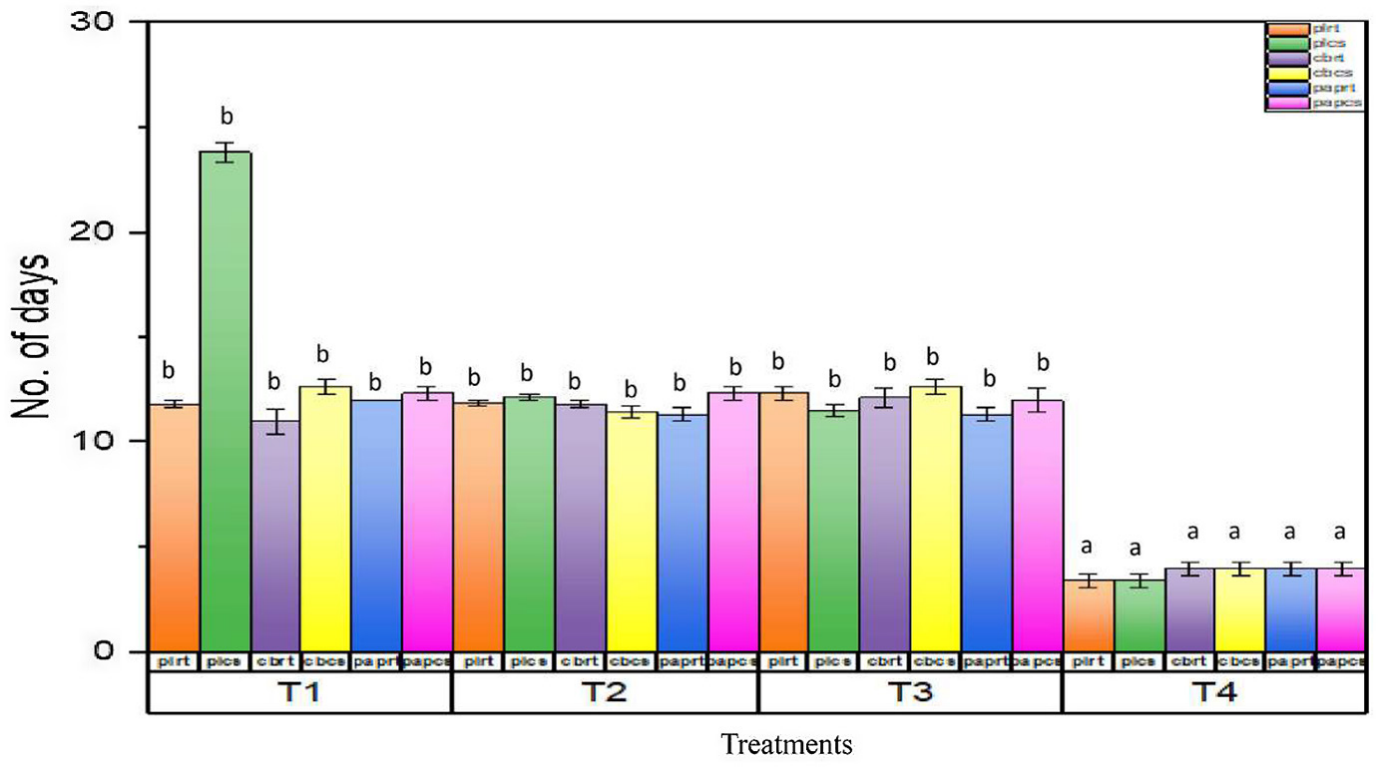
Shelf life of strawberry fruits during storage period. T1 (10 mM SeNPs); T2 (20 mM); T3 (30 mM); T4 (control); CBCS (cardboard packaging at 6°C); CBRT (cardboard packaging at 25°C); PLCS (plastic packaging at 6°C); PLRT (plastic packaging at 25°C); PAPCS (paper packaging at 6°C), and PAPRT (paper packaging at 25°C). Same letters on bars indicate the same difference between means, whereas different letters show different mean differences between mean values at *p* < .05 according to Duncan's multiple range test.

The effect of different parameters on the shelf life of strawberry fruits was observed and depicted in Figure 7. The length of the shelf life was calculated by observing the strawberry fruits after an interval of 4 days and then the treatments with no decay signs were allowed to proceed further, whereas the decayed fruits were discarded and the day of their decay was documented. In distilled water extract (T_4_) of all the packaging materials and temperatures, a very short shelf life period was observed, that is, between 1 and 4 days approximately. Significant increases in the shelf life of strawberry fruits were observed at 6°C. However, T1‐treated strawberry fruits stored at 6°C were observed marketable up to 12 days. However, a slight decrease in the moisture content was observed on day 20.

#### 3.4.3 Peroxidase (POD) activity

During storage period, peroxidase activity of strawberry fruits was observed. As given in Table 2, a decrease in POD activity was observed in control strawberry fruits. A significant increase in peroxidase activity was observed in strawberry fruits treated with SeNPs. Strawberry fruits treated with SeNPs stored at 6°C showed an increase in POD activity for up to 16 days, as compared with fruits stored at 25°C. The T1 (10 mM SeNPs) application on strawberry fruits was observed to increase the POD activity for up to the 16th day. Peroxidase decreases the production of reactive oxygen species and thus enhances the shelf life of fruits and vegetables (Huang et al., 2023).

**TABLE 2:** Peroxidase (POD) activity among all treatments in different packaging at cold/ room storage.

Peroxidase activity									
Storage condition	At 6°C						At 25°C		
Days	4	8	12	16	20	24	4	8	12
*Plastic packaging*									
T_1_	0.47 ± 0.05^d^	0.6 ± 0.005^e^	0.85 ± 0.03^f^	0. 9 ± 0^g^	0.24 ± 0.00^c^	0.24 ± 0.00^c^	0.23 ± 0.003^a^	0.21 ± 0.003^a^	0.24 ± 0.003^a^
T_2_	0.24 ± 0^c^	0.25 ± 0.017^c^	0.022 ± 0.0^a^	–	–	–	0.23 ± 0.003^a^	0.22 ± 0.005^a^	0.19 ± 0.003^a^
T_3_	0.2 ± 0^c^	0.21 ± 0^c^	0.19 ± 0.01^b^	–	–	–	0.16 ± 0.003^a^	0.17 ± 0.005^a^	0.16 ± 0.005^a^
T_4_	0.19 ± 0.003^b^	–	–	–	–	–	0.15 ± 0.005^a^	–	–
*Cardboard packaging*									
T_1_	0.26 ± 0^c^	0.26 ± 0^c^	0.28 ± 0^c^	–	–	–	0.16 ± 0^a^	0.21 ± 0.003^a^	0.19 ± 0.003^a^
T_2_	0.23 ± 0^c^	0.22 ± 0^c^	0.22 ± 0^c^	–	–	–	0.23 ± 0.003^a^	0.19 ± 0.003^a^	0.18 ± 0.005^a^
T_3_	0.2 ± 0^c^	0.20 ± 0^c^	0.18 ± 0.003^b^	–	–	–	0.15 ± 0.003^a^	0.17 ± 0.008^a^	0.17 ± 0.005^a^
T_4_	0.18 ± 0^b^	–	–	–	–	–	0.14 ± 0.005^a^	–	–
*Paper packaging*									
T_1_	0.24 ± 0^c^	0.23 ± 0.0^c^	0.21 ± 0.0^c^	–	–	–	0.23 ± 0.003^a^	0.21 ± 0.003^a^	0.32 ± 0.01^a^
T_2_	0.22 ± 0^c^	0.213 ± 0.005^c^	0.2 ± 0.00^c^	–	–	–	0.23 ± 0.003^a^	0.19 ± 0.003^a^	0.18 ± 0.003^a^
T_3_	0.18 ± 0^b^	0.16 ± 0^b^	0.15 ± 0.03^b^	–	–	–	0.17 ± 0.003^a^	0.16 ± 0.006^a^	0.11 ± 0.003^a^
T_4_	0.15 ± 0^b^	–	–	–	–	–	0.16 ± 0.003^a^	–	–

*Note*: T1 (10 mM SeNPs); T2 (20 mM SeNPs); T3 (30 mM SeNPs); T4 (control), PL (plastic packaging); CB (cardboard packaging); pap (paper packaging); CS (6°C); and RT (25°C). Letters in superscript indicate the same difference between means, whereas different letters show different mean differences between mean values at *p* < .05 according to Duncan's multiple range test.

### 3.5 Catalase (CAT) activity

Catalase (CAT) plays a vital role in neutralization by decomposition of H_2_O_2._ During storage period, catalase activity was observed and it is presented in Table 3. The CAT activity of the treated strawberry fruit in plastic packaging was generally higher in room storage (25°C) than that in cold storage (6°C) on day 4. Because at high temperature, the metabolism of fruit is fast, and low temperature slows down the metabolic process of fruits. Maximum CAT activity of 0.25 ± 0.01 was recorded in T_1_ stored in plastic packaging at 6°C on day 4 which increased to 0.45 ± 0.00 on day 12. An increasing trend in catalase activity during storage period was observed in SeNPs‐treated strawberry fruits stored at 6°C than the control treatments and the fruits kept at 25°C. In cold storage, the CAT activity was increased as the days of storage increased. Increase in the catalase activity of strawberry fruits up to 5 days during storage period was observed by Haider et al. (2022). The study checked the effect of application of *Eucalyptus* leaves extract on postharvest strawberry fruits.

**TABLE 3:** Catalase (CAT) activity among all treatments in different packaging at cold/room storage.

Catalase activity (μmol H_2_O_2_)									
Storage condition	At 6°C						At 25°C		
Days	4	8	12	16	20	24	4	8	12
*Plastic packaging*									
T_1_	0.25 ± 0.01^a^	0.33 ± 0.005^b^	0.42 ± 0.005^c^	0.54 ± 0^a^	0.023 ± 0.03^a^	0.24 ± 0.03^a^	0.2 ± 0.03^a^	0.18 ± 0.02^a^	0.19 ± 0.03^a^
T_2_	0.23 ± 0.003^a^	0.24 ± 0^a^	0.22 ± 0^a^	–	–	–	0.12 ± 0.003^a^	0.12 ± 0.03^a^	0.15 ± 0.03^a^
T_3_	0.21 ± 0.002^a^	0.22 ± 0^a^	0.19 ± 0.005^a^	–	–	–	0.13 ± 0.003^a^	0.13 ± 0.03^a^	0.12 ± 0.03^a^
T_4_	0.19 ± 0.002^a^	–	–	–	–	–	0.11 ± 0.002^a^	–	–
*Cardboard packaging*									
T_1_	0.2 ± 0^a^	0.21 ± 0^a^	0.19 ± 0^a^	–	–	–	0.05 ± 0.003^a^	0.12 ± 0.005^a^	0.08 ± 0.003^b^
T_2_	0.24 ± 0.005^a^	0.17 ± 0.005^a^	0.16 ± 0.002^a^	–	–	–	0.12 ± 0.003^a^	0.14 ± 0.003^a^	0.061 ± 0.003^b^
T_3_	0.15 ± 0^a^	0.13 ± 0.0057^a^	0.12 ± 0^a^	–	–	–	0.07 ± 0.003^b^	0.06 ± 0.003^b^	0.05 ± 0.003^a^
T_4_	0.12 ± 0.003^a^	–	–	–	–	–	0.05 ± 0.003^a^	–	–
*Paper packaging*									
T_1_	0.19 ± 0^a^	0.18 ± 0.003^a^	0.17 ± 0.005^a^	–	–	–	0.11 ± 0.003^a^	0.11 ± 0.003^a^	0.05 ± 0.01^a^
T_2_	0.15 ± 0.002^a^	0.16 ± 0^a^	0.15 ± 0.003^a^	–	–	–	0.19 ± 0.005^a^	0.07 ± 0.002^b^	0.06 ± 0.001^b^
T_3_	0.14 ± 0^a^	0.13 ± 0.003^a^	0.11 ± 0^a^	–	–	–	0.06 ± 0.001^b^	0.068 ± 0.0017^b^	0.049 ± 0.005^a^
T_4_	0.11 ± 0.03^a^	–	–	–	–	–	0.045 ± 0.005^a^	–	–

*Note*: T_1_ (10 mM SeNPs); T_2_ (20 mM SeNPs); T_3_ (30 mM SeNPs); and T_4_ (control). Letters in superscript indicate the same difference between means, whereas different letters show different mean differences between mean values at *p < 0.05* according to Duncan's multiple range test.

### 3.6 Total phenolic content and antioxidant activities

Tables 4, 5, 6 present the total phenolic content and antioxidant profiles of strawberry fruits packed in different packaging materials (plastic, cardboard, paper) over the course of 16 days in storage at 6°C and 25°C.

**TABLE 4:** Total phenolic contents among all treatments in different packaging at cold and room storage.

Total phenolic content (Gallic acid mg/g)									
Storage condition	At 6°C						At 25°C		
Days	4	8	12	16	20	24	4	8	12
*Plastic packaging*									
T_1_	328.7 ± 0.9^e^	323.7 ± 0.9^d,e^	323.7 ± 0.9^d,e^	320.2 ± 0^d^	310.9 ± 0^d^	291 ± 0^d^	111.3 ± 0.3^a^	112.2 ± 0.3^a^	120 ± 0.3^a^
T_2_	137.8 ± 0.3^b^	142.9 ± 0^c^	139.5 ± 0.9^b^	–	–	–	99 ± 0.57^a^	110.6 ± 0.3^a^	122 ± 0.5^a^
T_3_	127.8 ± 0.1	122.8 ± 0.17^a^	117.7 ± 0.2^a^	–	–	–	162.3 ± 0.3^a^	120.3 ± 0.3^a^	107.3 ± 0.3^a^
T_4_	117.6 ± 0.2^a^	–	–	–	–	–	60.6 ± 0.6^a^	–	–
*Cardboard packaging*									
T_1_	112.7 ± 0.2^a^	117.7 ± 0.2^a^	107 ± 0.8^a^	–	–	–	113.3 ± 0.3^a^	124.3 ± 0.3^a^	100 ± 0.3^a^
T_2_	97.8 ± 0.3^a^	91.8 ± 0.7^a^	87.4 ± 0.4^a^	–	–	–	129 ± 0.5^a^	124.3 ± 0.3^a^	99.3 ± 0.3^a^
T_3_	81.7 ± 0.6^a^	76.7 ± 0.6^a^	76.95 ± 0.5^a^	–	–	–	117.3 ± 0.3^a^	130 ± 0.3^a^	98.6 ± 0.3^a^
T_4_	72.3 ± 0.5^a^	–	–	–	–	–	100.6 ± 0.3^a^	–	–
*Paper packaging*									
T_1_	177.4 ± 0.5^b^	131.3 ± 0.2^a^	72.02 ± 0.02	–	–	–	121 ± 0.3^a^	132 ± 0.3^a^	131 ± 0.5^a^
T_2_	61.6 ± 0.5^a^	51.5 ± 0.4^a^	41.3 ± 0.2^a^	–	–	–	114.6 ± 0.8^a^	116 ± 1.20^a^	121.6 ± 0.3^a^
T_3_	36.3 ± 0.3^a^	31.3 ± 0.2^a^	31.45 ± 0^a^	–	–	–	119.3 ± 0.3^a^	122.3 ± 0.3^a^	99.3 ± 0.3^a^
T_4_	26.6 ± 0.3^a^	–	–	–	–	–	87.6 ± 0. 3^a^	–	–

*Note*: T_1_ (10 mM SeNPs); T_2_ (20 mM SeNPs); T_3_ (30 mM SeNPs); and T_4_ (control). Letters in superscript indicate the same difference between means, whereas different letters show different mean differences between mean values at *p* < .05 according to Duncan's multiple range test.

**TABLE 5:** DPPH radical scavenging activity among all treatments in different packaging at cold and room storage.

Scavenging (%)									
Storage condition	At 6°C						At 25°C		
Days	4	8	12	16	20	24	4	8	12
*Plastic packaging*									
T_1_	77.3 ± 0.5^e^	82.6 ± 0.4^f^	84.6 ± 0.5^f^	87.3 ± 0^f^	51 ± 0.6^d^	51.06 ± 0.6^d^	50.4 ± 0.4^d^	51.6 ± 0.3^d^	54.6 ± 0.3^d^
T_2_	51 ± 0.06^d^	53.7 ± 0.7^d^	54.4 ± 0.17^d^	–	–	–	52 ± 0.5^d^	51.1 ± 0.4^d^	47.8 ± 0.1^c^
T_3_	50.4 ± 0^d^	51.06 ± 0.6^d^	50.4 ± 0^d^	–	–	–	46.5 ± 0.5^b^	44.5 ± 0.28^b^	42.6 ± 0.3^b^
T_4_	47.4 ± 0^c^	–	–	–	–	–	43.6 ± 0.3^b^	±	±
*Cardboard packaging*									
T_1_	53.7 ± 0.7^d^	51.06 ± 0.6^d^	50.4 ± 0.17^d^	–	–	–	49.3 ± 0.6^c^	48.3 ± 0.6^c^	46 ± 0^c^
T_2_	42.4 ± 0.37^b^	41.2 ± 0^b^	40.5 ± 0.05^b^	–	–	–	48.2 ± 0.2^b^	44.6 ± 0.6^b^	44.6 ± 0.3^b^
T_3_	37 ± 0^a^	36 ± 0^a^	34 ± 0.2^a^	–	–	–	43.3 ± 0.3^b^	43.6 ± 0.3^b^	41.3 ± 0.3^b^
T_4_	40.4 ± 0.5^b^	–	–	–	–	–	40.6 ± 0.6^b^	–	–
*Paper packaging*									
T_1_	50.4 ± 0.1^d^	52.4 ± 0.17^d^	47 ± 0.5^c^	–	–	–	47 ± 0.5^c^	43.5 ± 0.3^b^	45.3 ± 0.3^b^
T_2_	41.4 ± 0.3^b^	40.2 ± 0^b^	39.5 ± 0.05^a,b^	–	–	–	46.3 ± 0.3^c^	45.3 ± 0.3^c^	45.6 ± 0.3^c^
T_3_	36 ± 0^a^	35 ± 0.5^a^	33 ± 0^a^	–	–	–	45.3 ± 0.3^c^	33.3 ± 0.3^a^	33.6 ± 0.3^a^
T_4_	33 ± 0^a^	–	–	–	–	–	22.3 ± 0.3^a^	–	–

*Note*: T_1_ (10 mM SeNPs); T_2_ (20 mM SeNPs); T_3_ (30 mM SeNPs) and T_4_ (control). Same letters on bars indicate the same difference between means, whereas different letters show different mean differences between mean values at *p* < .05 according to Duncan's multiple range test.

**TABLE 6:** Total antioxidant activity among all treatments in different packaging at cold and room storage.

Total antioxidant activity (*ƛ*_max_ 695 nm)									
Storage condition	At 6°C						At 25°C		
Days	4	8	12	16	20	24	4	8	12
*Plastic packaging*									
T_1_	0.5 ± 0.005^f^	0.57 ± 0.01^f^	0.76 ± 0.01^g^	0.82 ± 0.11^h^	0.12 ± 0.01^e^	0.12 ± 0.011^/e^	0.08 ± 0.003^d^	0.007 ± 0.003^c^	0.06 ± 0.003^c^
T_2_	0.12 ± 0.01^e^	0.14 ± 0.00^e^	0.05 ± 0^c^	–	–	–	0.06 ± 0.01^c^	0.07 ± 0.01^c^	0.07 ± 0.01^c^
T_3_	0.1 ± 0.001^e^	0.09 ± 0.03^d^	0.03 ± 0.01^a^	–	–	–	0.05 ± 0.008^c^	0.03 ± 0.005^a^	0.03 ± 0.003^a^
T_4_	0.08 ± 0^d^	–	–	–	–	–	0.03 ± 0.09^a^	±	±
*Cardboard packaging*									
T_1_	0.12 ± 0^e^	0.13 ± 0.05^e^	0.11 ± 0^e^	–	–	–	0.05 ± 0.01^c^	0.06 ± 0.002^c^	0.07 ± 0.0003^c^
T_2_	0.09 ± 0.01^d^	0.08 ± 0^d^	0.06 ± 0.02^c^	–	–	–	0.06 ± 0.02^c^	0.7 ± 0.0003^c^	0.04 ± 0.002^b^
T_3_	0.06 ± 0.01^c^	0.05 ± 0^d^	0.06 ± 0.01^c^	–	–	–	0.03 ± 0.0011^a^	0.038 ± 0.001^a^	0.04 ± 0.003^a^
T_4_	0.04 ± 0.001^b^	–	–	–	–	–	0.02 ± 0.003^a^	–	–
*Paper packaging*									
T_1_	0.1 ± 0^e^	0.09 ± 0^d^	0.07 ± 0.005^d^	–	–	–	0.06 ± 0.003	0.04 ± 0.002^a^	0.05 ± 0.003
T_2_	0.05 ± 0^c^	0.06 ± 0.005^d^	0.07 ± 0.005^d^	–	–	–	0.06 ± 0.0005^c^	0.054 ± 0.0003^c^	0.046 ± 0.001^a^
T_3_	0.05 ± 0^c^	0.04 ± 0.01^a^	0.01 ± 0^a^	–	–	–	0.04 ± 0.001^b^	0.038 ± 0.001^a^	0.036 ± 0.003^a^
T_4_	0.03 ± 0.01^a^	–	–	–	–	–	0.042 ± 0.001^b^	±	±

*Note*: T_1_ (10 mM SeNPs); T_2_ (20 mM SeNPs); T_3_ (30 mM SeNPs) and T_4_ (control). Same letters on bars indicate the same difference between means, whereas different letters show different mean differences between mean values at *p* < .05 according to Duncan's multiple range test.

Table 4 shows the total phenolic content of strawberry fruits during storage period. The total phenolic content of fresh strawberry fruit was calculated as 121 ± 2 (ga mg/g). A significant decrease in the total phenolic content of stored fruits was observed in control strawberry fruits at all the storage conditions. During storage, TPC values of T1, T2, and T3 showed a tendency to increase from day 4, where the T1 samples stored in plastic packaging at 6°C showed higher TPC values than the initial value. Nassarawa et al. (2022) documented that the red light‐treated grapes maintained higher levels of total phenolic content as compared with control during storage period. Phenolic compound is an essential constituent of plants with redox properties and is responsible for the antioxidant activity. Loss of the antioxidant activity is due to the oxidation of the phenolic compounds as a result of high temperatures and exposure to light.

Two methods (total antioxidant assay and DPPH free radical scavenging assay) were performed to check the antioxidant capacity of strawberry fruits during storage period. An increasing trend in antioxidant activities was observed in SeNPs‐treated strawberry fruits during storage period than the control at the end of the storage. Strawberry fruits treated with 10 mM SeNPs stored at 6°C in plastic packaging were observed to have higher scavenging (%) that is, 87.33% and TAA on day 16 than the initial value. Total phenolic content and increase in antioxidant could be associated with the postharvest treatment's efficiency to scavenge reactive oxygen species, and as a consequence, oxidative damage to the fresh commodities reduces (Lo'ay & El‐Ezz, 2021). An increase in antioxidant activities of postharvest grape fruit was also documented during storage period by Nassarawa et al. (2022). In the present study, cold storage conditions significantly increased the total phenolics, which can be attributed to changes occurring in phenol metabolism during storage. The decrease in the total phenolic capacity is attributed to phenolic degradation as a result of enzymatic activities occurring due to high temperature (El‐Gioushy et al., 2022).

### 3.7 Relationship of strawberry fruit characteristics

Pearson's correlation coefficient test was applied to investigate the inter‐correlation of strawberry fruits’ quality attributes during storage period (Table 7). Both positive and negative significant correlations were found among fruit traits (physiological weight loss, moisture content, physiological decay loss, peroxidase, catalase, total phenolic content, DPPH, and Total antioxidant activity). Physiological weight loss is positively correlated with moisture content (*r* ^2^ = 0.753). Maintenance of moisture content maintains the weight of the fruits. A decrease in moisture content reduces the weight of the fruits. Physiological decay loss was found to be negatively correlated with moisture content. A decrease in moisture content increases the decay percentage. Positive correlation (*r* ^2^ = 0.95) was observed between moisture content and peroxidase activity suggesting that the moisture content lowers the production of reactive oxygen species. DPPH radical scavenging activity and total antioxidant activity are positively correlated with the total phenolic content of the fruits. The results are endorsed by the findings of Khedr (2022).

**TABLE 7:** Pearson correlation of the Strawberry fruits of different parameters

	PWL	PDL	MC	POD	CAT	TPC	DPPH	TAA
PWL	1							
PDL	−0.34*	1						
MC	0.753*	−0.260*	1					
POD	0.852*	−0.591*	0.95*	1				
CAT	0.632*	−0.591*	0.359*	0.98*	1			
TPC	0.744*	−0.617*	0.31*	0.772*	0.772*	1		
DPPH	0.811*	−0.582*	0.255*	0.784*	0.784*	0.884*	1	
TAA	0.855*	−0.617*	0.290*	0.836*	0.836*	0.836*	0.889**	1

*Note*: *2 tailed* significance test is performed. Asterick sign * depicts that correlation is significant at *p* < .05.

Abbreviations: CAT, catalase; DPPH, Diphenyl Picryl Hydrazyl Radical; MC, moisture content; PDL, physiological decay loss; POD, Peroxidase; PWL, physiological weight loss; TAA, total antioxidant activity; TPC, total phenolic content.

Pearson correlation coefficient test was also performed to check the effect of each treatment on strawberry fruits’ quality attributes during storage period (Table 8a–d). Among all the treatments, control treatment showed non‐significant relationship among quality attributes of strawberry fruits during storage period as compared with other treatments. However, T1 application was observed to have significant negative and positive correlations among quality attributes of strawberry fruits as compared with other treatments. The study confirmed the relationship of moisture content with physiological weight loss and physiological decay loss. Phenolic compounds scavenge reactive oxygen species; therefore, are known as antioxidants. They play an important role in the defense mechanism of fruits and vegetables. Total phenolic content protects plants from pathogen invasion (El‐Gioushy et al., 2022). Based on these findings, we can hypothesize that phenolic compounds increase the antioxidant capacity of strawberry fruits during storage period. Nanotechnology provides many benefits to the food industry in improving food taste, texture, sensitivity, quality, shelf health, and food safety. Nanotechnology is used in food industries for preparation, packaging, marketing, and for better yield (Nile et al., 2020). Likewise, in the present study, it has been confirmed that the strawberry fruits treated with selenium nanoparticles showed greater shelf life as compared with the non‐treated ones.

**TABLE 8:** (a) Effect of T1 (10 mM SeNPs) on strawberry fruits characteristics during storage period. (b) Effect of T2 (20 mM SeNPs) on strawberry fruits characteristics during storage period. (c) Effect of T3 (30 mM SeNPs) on strawberry fruits characteristics during storage period. (d) Effect of T4 (control) on strawberry fruits characteristics during storage period.

	PWL	MC	PDL	POD	CAT	DPPH	TAA	TPC
(a)								
T1CS								
PWL	1							
MC	0.93*	**1**						
PDL	−0.93*	−0.832*	**1**					
POD	0.406	0.408	−0.30	**1**				
CAT	0.738*	0.791*	−0.581	0.778*	**1**			
DPPH	0.777*	0.796*	−0.663*	0.57	0.779*	**1**		
TAA	0.817*	0.890*	−0.633*	0.66*	0.96*	0.845*	**1**	
TPC	0.820*	0.914*	−0.628	0.482	0.860*	0.835*	0.959*	**1**
**T1RT**								
PWL	1							
MC	−0.272	1						
PDL	0.745*	−0.2769	1					
POD	−0.107	−0.358	−0.203	1				
CAT	−0.211	0.403	0.059	−0.59	1			
DPPH	−0.607	0.6135	−0.565	0.270	−0.150	1		
TAA	−0.565	0.369	−0.69*	0.34	−0.510	0.805*	1	
TPC	−0.135	0.745	−0.198	−0.375	0.612	0.262	0.002	1

	PWL	MC	PDL	POD	CAT	DPPH	TAA	TPC
(b)								
*T2CS*								
PWL	1							
MC	−0.611	1						
PDL	0.673*	−0.761*	1					
POD	−0.251	0.332	−0.548	1				
CAT	−0.548	0.882*	−0.423	0.167	1			
DPPH	−0.702*	0.472	−0.363	0.437	0.576	1		
TAA	−0.316	0.256	−0.147	0.717*	0.376	0.719*	1	
TPC	−0.299	0.368	−0.10	0.55	0.525	0.7*	0.955*	1
*T2RT*								
PWL	1							
MC	−0.345	1						
PDL	0.832*	−0.358	1					
POD	−0.071	0.333	0.131	1				
CAT	−0.847*	0.643	−0.80*	−0.02	1			
DPPH	−0.909*	0.174	−0.687*	0.203	0.68*	1		
TAA	−0.613	0.893*	−0.716*	0.22	0.8*	0.395	1	
TPC	−0.220	0.944*	−0.30709	0.338	0.497	−0.013	0.859*	1

	PWL	MC	PDL	POD	CAT	DPPH	TAA	TPC
(c)								
*T3CS*								
PWL	1							
MC	−0.546	1						
PDL	0.924*	−0.63895	1					
POD	−0.31	0.55066	−0.32085	1				
CAT	−0.410	0.860*	−0.46927	0.33966	1			
DPPH	−0.284	0.78*	−0.33498	0.36774	0.974*	**1**		
TAA	−0.443	0.905*	−0.54386	0.46924	0.979*	0.957*	**1**	
TPC	−0.159	0.863*	−0.3033	0.52231	0.878*	0.890*	0.917*	1
*T3RT*								
PWL	1							
MC	−0.303	1						
PDL	0.759*	−0.218	1					
POD	−0.820*	0.144	−0.910*	1				
CAT	−0.511	0.828*	−0.433	0.306	1			
DPPH	−0.662	0.207	−0.829*	0.645	0.598	1		
TAA	−0.290	0.187	−0.23	0.259	0.516	0.476	1	
TPC	0.130	0.641	0.151	−0.382	0.50	−0.023	−0.347	1

	PWL	MC	PDL	POD	CAT	DPPH	TAA	TPC
(d)								
*T4CS*								
PWL	1							
MC	−0.932	1						
PDL	0.394	−0.035	1					
POD	−0.99*	0.939	−0.376	1				
CAT	−0.59	0.847	0.5	0.614	1			
DPPH	0.394	−0.035	1	−0.376	0.5	1		
TAA	−0.59	0.847	0.5	0.614	1	0.5	1	
TPC	−0.994	0.887	−0.491	0.991	0.508	−0.491	0.508	1
*T4RT*								
PWL	1							
MC	−0.947	1						
PDL	−0.747	0.494	1					
POD	−0.322	0.0024	0.870	1				
CAT	−0.966	0.832	0.893	0.556	1			
DPPH	−0.966	0.832	0.893	0.556	1	1		
TAA	−0.706	0.896	0.057	−0.441	0.5	0.5	1	
TPC	0.602	−0.826	0.080	0.560	−0.375	−0.375	−0.99	1

*Note*: *2 tailed* significance test is performed. Asterick sign * depicts that correlation is significant at *p <* 0.05.

Abbreviations: CAT, catalase; DPPH, Diphenyl Picryl Hydrazyl Radical; MC, moisture content; PDL, physiological decay loss; POD, Peroxidase; PWL, physiological weight loss; T1CS, 10 mM SeNPs ± 6°C; T2CS, 20 mM SeNPs at ±6°C; T3CS, 30 mM SeNPs at ±6°C; T4CS, control at ±6°C; T1RT, control at ±25°C; T2RT, 20 mM SeNPs at ±25°C; T3RT, 30 mM SeNPs at ±25°C; T4RT, control at ±25°C; TAA, total antioxidant activity; TPC, total phenolic content.

All the bold values showed significant correlation among different variables.

### 3.8 Microbial susceptibility analysis

T_1_ (10 mM) of selenium nanoparticles sprayed at strawberry fruit stored in plastic packaging under cold storage have shown a zone of inhibition (mm) against different bacterial and fungal strains. The zone of inhibition was measured with the help of a millimeter scale, from one edge to the other edge. The zones shown as treated strawberry fruits were compared with the zones produced by standard antimicrobial drugs, that is, ampicillin for bacteria and Fluconazole for fungus.

Antibacterial activity of strawberry fruits during storage period was observed by the agar well diffusion method. Treated strawberries with prolonged shelf life exhibited a zone of inhibition against *Pseudomonas fluorescence* and *Bacteroides ovatus*. May and Fickak (2003) evaluated that *Pseudomonas* sp. was the prime origin of decaying of postharvested crops when kept in a refrigerator, but in a recent study, it was observed that the treated strawberries have developed a potentiality to suppress the growth of *Pseudomonas fluorescence* (Table 9). Zones of inhibition (mm) were measured against *Pseudomonas fluorescence* and *Bacteroides ovatus* and compared with the zones formed by Ampicillin. Ampicillin developed a zone of inhibition of 33 ± 0.1 mm against *B. ovatus* and 28 ± 0.3 against *P. fluorescence*. Fresh strawberry fruits exhibited 30 ± 0.3 mm zone of inhibition against *P. fluorescence* and 27 ± 0.3 against *B. ovatus* at the highest concentration. However, on 16th day, strawberry fruits were observed to have 20 ± 1.1 against *P. fluorescence* and 35 ± 1 against *B. ovatus*. Antifungal potential of strawberry fruits was also observed and compared with the standard drug (Flucanazole) (Table 10). Flucanazole zones of inhibition were observed as 34 ± 0.3 against *Fusarium oxysporum* and 31 ± 0.3 against *Aspergillus flavus*. On day 1 of storage, 15.5 ± 1 mm against *Fusarium oxysporum* and 13 ± 0.5 against *Aspergillus flavus* zones of inhibition were observed. On day 16 of storage, 9 ± 1 mm zone of inhibition against *F. oxysporum* and 10 ± 0.5 mm against *A. flavus* was measured. Liya and Siddique (2018) study endorsed the results. Their study has reported that the ethanolic extracts of strawberry fruits exhibit antibacterial potential against *Enterococcus faecalis* (ATCC: 29212), *E. coli* (ATCC: 25922), *Pseudomonas aeruginosa* (ATCC: 27853), *E. coli* (ATCC: 15922), and *Klebsiella pneumonia*. Terry et al. (2004) stated that strawberry fruits possess antifungal activity because of the presence of phenolic compounds.

**TABLE 9:** Zone of Inhibition (mm) shown by different bacteria against various treatments.

Zone of Inhibition (mm)						
Conc.	*Bacteriodes ovatus*			*Pseudomonas fluorescence*		
	10^−1^	10^−2^	10^−3^	10^−1^	10^−2^	10^−3^
Fresh Strawberry fruits	27.3 ± 0.3^k^	12.3 ± 0.3^c^	8.3 ± 0.3^b^	30 ± 0.3^l^	24.3 ± 0.3^j^	14 ± 0.3^e^
T_1_ PLCS (16th day)	20 ± 0.3^g^	18 ± 0.3^f^	9.3 ± 0.3^b^	34.6 ± 0.3^n^	22.3 ± 0.3^h^	8 ± 0.3^b^
Ampicillin	33 ± 0.1^m^	23.3 ± 0.3^h,i^	13.6 ± 0.3^c,d^	28 ± 0.3^k^	15 ± 1.0^e^	5.5 ± 0.1^a^

**TABLE 10:** Zone of Inhibition (mm) shown by fungal strains against different treatments.

Zone of Inhibition (mm)						
Conc.	*Fusarium oxysporum*			*Aspergillus flavus*		
	10^−1^	10^−2^	10^−3^	10^−1^	10^−2^	10^−3^
Fresh Strawberry fruits	15.5 ± 0.3^c,d^	–	–	13 ± 0.5^c^	–	–
T_1_ PLCS (16th day)	9 ± 0^a^	–	–	10 ± 0.5^b^	–	–
Flucanazole	34 ± 0.3^g^	30.3 ± 0.3^f^	21 ± 0.1^e^	31 ± 0.1^f^	25 ± 0.6^e^	15 ± 0.3^c,d^

## 4 CONCLUSION

The results of the recent study indicate that the green synthesized SeNPs possess antioxidant and antimicrobial potential. Green synthesized SeNPs application on postharvest strawberry fruits increases the shelf life, keeps up the quality attributes, and enhances peroxidase activity, catalase activity, total phenolic content, and antioxidant capacity.

## ACKNOWLEDGEMENT

In this research work special acknowledgement goes towards research center of the Future University in Egypt. Princess Nourah bint Abdulrahman University Researchers Supporting Project number (PNURSP2023R155), Princess Nourah bint Abdulrahman University, Riyadh, Saudi Arabia.

## CONFLICT OF INTEREST STATEMENT

The authors confirm that they have no conflicts of interest with respect to the work described in this manuscript.

## Data Availability

The data that support the findings of this study are available from the corresponding author upon reasonable request.
